# Simultaneous bilateral ulnar neuropathy: an unusual complication caused by neuroleptic treatment-induced tardive dyskinesia: A Case Report

**DOI:** 10.1097/MD.0000000000017863

**Published:** 2019-11-11

**Authors:** Yi-Ping Wei, Shan-Wei Yang

**Affiliations:** Department of Orthopedics, Kaohsiung Veterans General Hospital, Taiwan, ROC.

**Keywords:** antipsychotic drug, paralysis, tardy ulnar nerve palsy, ulnar neuropathy

## Abstract

**Rationale:**

In the past decade, only a few studies have focused on simultaneous bilateral ulnar neuropathy.

**Patient concerns:**

A 54-year-old Asian male who has suffered from paranoid schizophrenia for 2 years. He reported that flexion contracture occurring over his fourth and fifth fingers on both hands appeared since six months after he started taking the antipsychotic drug. The electromyogram revealed bilateral ulnar neuropathy with chronic axonal degeneration at the elbow level. McGowan classification was performed to evaluate the severity of the ulnar nerve injury, and the patient was diagnosed with a grade 3 injury on his left hand and a grade 2 injury on his right hand.

**Diagnosis:**

Simultaneous bilateral ulnar neuropathy at the elbow, a complication caused by tardive dyskinesia in a patient under the high-dose, first-generation, antipsychotic drug.

**Interventions:**

We consulted a psychiatrist to assist in adjusting the patient's kind of the antipsychotic drug and performed the anterior transposition of ulnar nerve to avoid nerve entrapment caused by tardive dyskinesia.

**Outcomes:**

Numbness of the palms continued to regress over the following 6 months after the anterior transposition of the ulnar nerve. Regression of the involuntary movements, including repeated bending of the elbows, and shaking of both feet, was noted from the patient but was incomplete.

**Lessons:**

Two literatures concluded that parkinsonian rigidity is the main cause of simultaneous bilateral ulnar neuropathy by Sampath et al and Kurlan et al. Unlike the cases of stereotyped posture-caused neural compression reported previously, we inferred that repeated involuntary motion caused by first-generation antipsychotic drug might have been one of the causes of the patient's nerve compression.

## 1 Introduction

CTS and UN at the elbow are the most 2 common nerve entrapment syndromes that plague the upper limbs.^[1,2]^ Typically, tardy ulnar nerve palsy occurs as a consequence of non-union of lateral condyle in child resulting in cubitus valgus deformity which ultimately is the cause of ulnar nerve palsy. In the past decade only a few studies have focused on simultaneous bilateral UN.^[3–5]^ Bilateral lesions are still considered to be associated with systemic diseases.^[3]^ Occupations associated with repeated physical labor may result in UN due to direct pressure on the ulnar nerve for those employees working with flexed elbows.^[4]^

In our case report, we present a rare case of simultaneous bilateral UN, caused by involuntary repetitive elbow flexion. Based on this investigation, we realized that a careful examination of a patient's medication history before making diagnosis is important.

## 2 Case report

We report the case of a 54-year-old Asian male who has suffered from paranoid schizophrenia for 2 years and who has multiple persecutory delusions and paranoid ideation. This patient compulsorily admitted to a psychiatric ward on the basis of extremely disturbing behaviors once. Oral Haloperidol has been prescribed at that time, and the patient responded to the medication very well. The daily dose of oral Haloperidol was increased to 15 mg since one year ago.

Due to the use of a high-dose, first-generation antipsychotic drug, extrapyramidal syndromes afflicted the patient. According to the patient's self-statement, akathisia symptoms—restlessness, anxiety, and agitation—began to appear 1 month after he started taking the antipsychotic drug. Moreover, other symptoms of tardive dyskinesia—including tongue protrusion, lip smacking, excessive eye blinking, repeated bending and straightening of the fingers and elbows, involuntary shaking of both feet—occurred suddenly at the time of the fourth months of his antipsychotic drug treatment and worsened gradually. The patient reported that he felt enchanted and was unable to control his body movements. As a result, Trihexyphenidly 5 mg twice a day for oral use was prescribed to treat the patient's extrapyramidal symptoms.

**Figure 1:**
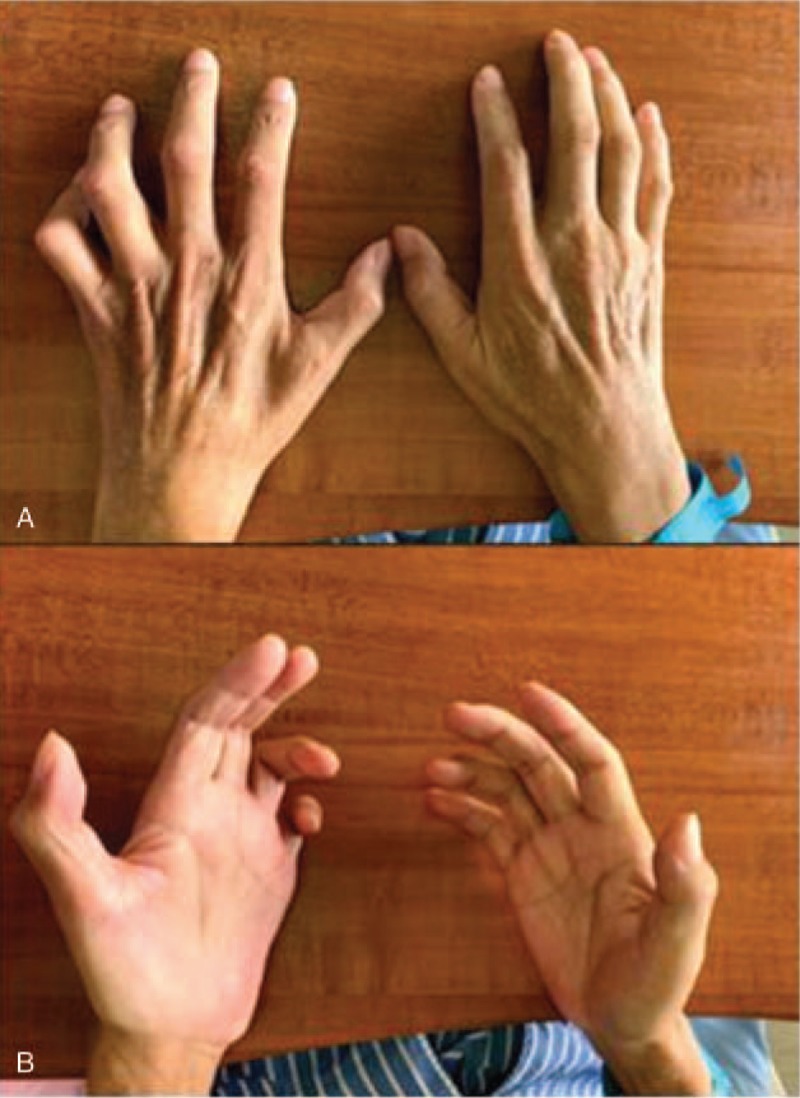
Benediction posture. The deformity results from a combination of adduction weakness (interossei muscle atrophy), and 4th and 5th clawing finger.

The patient also reported that flexion contracture occurring over his fourth and fifth fingers on both hands appeared since six months after he started taking the antipsychotic drug. The symptoms worsened very quickly to the point that he was unable to straighten his fourth and fifth fingers (Fig. 1). Because of the rapid loss of the function of his hands, he came to our orthopedic clinic for help. Physical examinations revealed that his fourth and fifth fingers were numb and his palmar muscle was characterized by marked atrophy. The EMG revealed bilateral UN with chronic axonal degeneration at the elbow level (Table 1). McGowan classification was performed to evaluate the severity of the ulnar nerve injury, and the patient was diagnosed with a grade 3 injury on his left hand and a grade 2 injury on his right hand.

**Table 1:**
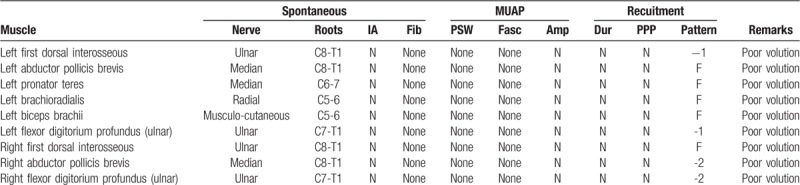
EMG summary of our case.

We examined the patient's past medical history to determine the cause of the bilateral UN. The patient reported body mass index (BMI): 20.54, no prior history of smoking, diabetes, gouty arthritis, trauma history, other joint pain or joint deformity as symptoms of rheumatoid arthritis. The patient was also free of hypothyroidism or a related metabolic disease, and he had no special family history. With the regard to medication use, he only took the drug prescribed by the psychiatric clinic. The patient had retired 6 years ago from selling clothing; he did not carry heavy loads or do other labor since then.

X-rays of the lateral and cubital tunnel view and anterior-posterior (AP) view revealed no obvious bone spurs and fractures, and no lumps or soft tissue masses were found upon palpation of the patient's elbows. A physical examination revealed a normal range of motion of elbows, negative Tinel signs at the wrists and positive at the elbows. However, we observed that the patient's elbow involuntarily underwent bending and straightening. After a surgical exploration for the neurolysis, we examined the ulnar nerve and its peripheral region. Neither the presence of nerve compression by adhesion band or nerve swelling was found. We strongly suspected that the bilateral UN in this patient was a complication caused by tardive dyskinesia, induced by antipsychotic drugs. As a result, psychiatrist consultation was done for making medication assessments and adjusting the patient's medications.

According to the patient on the telephone access, numbness of the palms continued to regress over the following 6 months after Haloperidol discontinuation and the anterior transposition of the ulnar nerve. However, the remaining ulnar claw deformities need longer time to observe the outcome. Regression of the involuntary movements, including repeated bending of the elbows, and shaking of both feet, was noted from the patient but was incomplete.

## 3 Discussion

In Table 2, we summarized the causes reported of the bilateral ulnar nerve neuropathy at the elbow.^[1,6–9]^

**Table 2:**
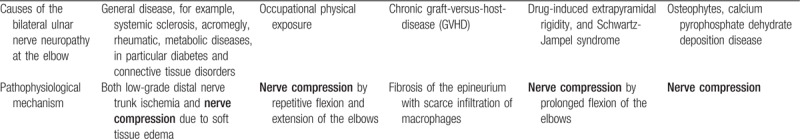
Causes and pathophysiological mechanism of the bilateral ulnar nerve neuropathy at the elbow.

Sampath et al (1997) explored the cause of antipsychotic-associated UN.^[5]^ Bilateral elbow flexion is one of the earliest signs of parkinsonian rigidity. Nerve compression caused by prolonged flexion posture is considered to be the primary cause of simultaneous bilateral UN. Kurlan et al^[10]^ published a case series focused on three patients with Parkinson's disease and on-off motor fluctuations that developed severe compression neuropathy following a sudden onset off period. These 2 literatures concluded that antipsychotic-associated rigidity is the main cause of UN. Robertson et al^[11]^ also emphasized prolonged elbow flexion puts a tremendous stretch on the nerve and simultaneously changes the diameter of the nerve, compressing it. So, flexion of the elbow for prolonged periods can also lead to ulnar neuropathy.

Today, it is very rare to find a patient who is prescribed large doses of a first-generation antipsychotic drug for a long period of time. Our patient suffered from obvious involuntary movements of his extremities. Repeated bending and straightening of his elbows may be one of the symptoms of tardive dyskinesia. The patient reported that his involuntary movements suddenly occurred 4 months after he began taking the antipsychotic drug. After 2 to 3 months, the flexion contracture over the patient's fourth and fifth fingers on both hands appeared. The onset timing of the disease could be logically inferred. Unlike the case of stereotyped posture-caused neural compression reported previously,^[5,10]^ we inferred that repeated involuntary motion might have been 1 of the causes of the patient's nerve compression. Similarly to our case, by Luigi Vimercati et al^[7]^ the profession of pizza chef exposing workers to several biomechanical risk factors (including repetitive and forceful movements of the hands and the elbows) for CTS and UN at the elbow was reported. Repetitive elbow flexion causes the ulnar nerve to be stretched, which compresses the nerve with in the cubital tunnel. During the elbow flexion at 45 degrees, the flexor carpi ulnaris aponeurosis stretches with 5 mm in length, closing down and further narrowing the cubital tunnel. The entrapment site is located 5 to 7 cm distal to the medial epicondyle. This anatomical consideration is the etiology and physiopathology of the UN at the elbow.^[11]^

### 3.1 Differential diagnosis

When simultaneous bilateral UN is diagnosed, the past history should focus on the predisposing factors: metabolic problems and systemic diseases such as diabetes mellitus, alcoholism, vitamin deficiency, anemia, neurological diseases with polyneuropathies. Besides, direct compression, such as occupation or personal habits including frequent leaning on the elbow, may result in the cubital tunnel compression syndrome of the dominant hand or bilateral hands.^[12]^

Our patient was free of the related metabolic diseases and systemic diseases. At the following 6 months, numbness of the palms continued to regress and regression of the involuntary movements, was noted. So, we strongly suspected that his simultaneous bilateral UN attributed to the involuntary movements of his upper extremities. The unusual case provided clinicians another different diagnosis for UN.

Besides, upper extremity involvement is unusual for peripheral neuropathies, in which lower extremity typically predominate; thus, the onset of upper extremity involvement may be a clue suggesting a tardive dyskinesia- induced peripheral neuropathy. Sampath et al^[5]^ described that prolonged fallen posture with the arms flexed (one of the earliest signs of parkinsonian rigidity) following the fluphenazine decanoate injection was the cause of bilateral UN. NSAID- induced peripheral neuropathy with “double crush” phenomenon was described by Rothenberg et al.^[13]^ Double crush phenomenon means that an individual nerve is more susceptible to entrapment if a generalized neuropathy exists.^[13]^

### 3.2 Drug induced bilateral ulnar nerve palsy

In the past decade, Rothenberg et al and Sampath et al had focused on medication induced bilateral UN (Table 3.)^[5,13]^ Rothenberg et al (1987) describe a patient with psoriatic arthritis, who developed a peripheral neuropathy (bilateral CTS, left cubital tunnel syndrome, and sural nerve) while taking hydroxychloroquine and naproxen. They identified naproxen, rather than hydroxychloroquine, as the cause of her neuropathy because of an exacerbation of the findings upon rechallenge with this drug.^[13]^ Sampath et al disclosed a distressing complication of drug-induced extrapyramidal rigidity and emphasized a serious pitfall of bilateral ulnar nerve paralysis. The finding of these previous studies clearly show that simultaneous bilateral UN must be considered about drug related side effect.

**Table 3:**
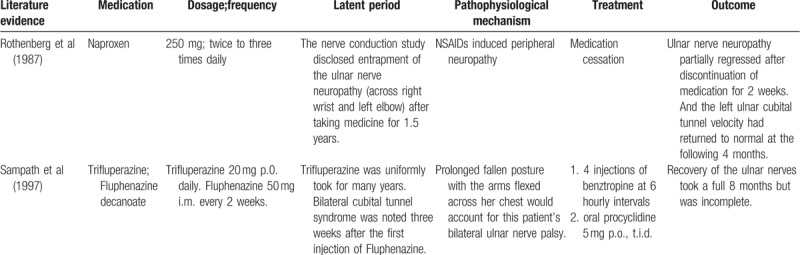
Literature evidence of medication induced bilateral ulnar neuropathy.

### 3.3 The advantages of nerve transposition

The advantages of decompression with anterior transposition for the ulnar nerve are that the nerve is moved to a new bed that may have less constrictive tissue and the nerve is effectively lengthened by a few centimeters after the anterior transposition. Recovery of the tardive dyskinesia was expected to take several months to several years, so we arranged anterior transposition for this patient to preserve his remaining ulnar nerve function and relieve the tension on the nerve.^[14]^

We did not observe nerve swelling during the surgical exploration because the acute inflammation phase was passing. About this patient, the UN had persisted for over 6 months before he visited our clinic visit. Although we consulted a psychiatrist to assist in adjusting the patient's kind of the antipsychotic drug and performed neurolysis for the patient, the expected recovery may be poor.

## Acknowledgments

The authors would like to thank Dr. Katherine K. for the English language review and Dr. Yang for the details of the antipsychotic drug.

## Author contributions

**Conceptualization:** Shan Wei Yang.

**Data curation:** Shan Wei Yang.

**Formal analysis:** Shan Wei Yang.

**Supervision:** Shan Wei Yang.

**Visualization:** Yi Ping Wei.

**Writing – original draft:** Yi Ping Wei.

**Writing – review & editing:** Shan Wei Yang.
